# Neurodevelopmental Disorders and Agricultural Pesticide Exposures: Shelton and Hertz-Picciotto Respond

**DOI:** 10.1289/ehp.1409124R

**Published:** 2015-04-01

**Authors:** Janie F. Shelton, Irva Hertz-Picciotto

**Affiliations:** 1 Independent consultant, Vienna, Austria; 2 University of California, Davis, Davis, California, USA

Burns et al. (2015) question whether residential proximity to agricultural pesticide applications can serve as a surrogate for actual exposures in research on autism spectrum disorders (ASDs) and neurodevelopmental delay. Previous work has consistently demonstrated that pesticide drift results in elevated levels of these compounds in both indoor air and house dust in residences located near agricultural applications (Fenske et al. 2002; Gunier et al. 2011; Harnly et al. 2005; Ward et al. 2006; Wofford et al. 2014). For example, Wofford et al. (2014) intensively monitored ambient air concentrations for 40 active ingredients or degradation products of pesticides in a California Central Valley city. Air concentrations of the organophosphates chlorpyrifos, diazinon, phosmet, and malathion increased after recent applications within 8 km of the city boundary. Notably, these associations were found despite mitigating factors such as those named by Burns et al. (2015), for example, weather conditions, wind direction, type of formulation, and application method. The temporal correspondence between applications and measured concentrations in air was particularly strong for chlorpyrifos (Wofford et al. 2014), which we found to be associated with an elevated prevalence of ASDs among children exposed *in utero* (Shelton et al. 2014).

Burns et al. (2015) also assert that levels reaching homes are inadequate to induce adverse effects on the fetus. In fact, between 1996 and 2008 pesticide drift or off-target spraying was associated with 2,945 cases of acute pesticide illness in 11 U.S. states, of which 14% were children under 15 years of age (Lee et al. 2011). Furthermore, one-third of acute pesticide illnesses occurring in U.S. schools in 1998–2002 were attributed to drift exposure from farmland (Alarcon et al. 2005). Thus, considerable evidence shows biologically harmful exposures can and do occur in areas surrounding agricultural fields where pesticides are applied.

With chlorpyrifos detectable in 70.5% of pregnant mothers living in an agricultural area in California (Huen et al. 2012), fetal exposure is surely widespread. Because the fetus cannot metabolize organophosphate chemicals as well as its adult mother can (Chen et al. 2003; Furlong et al. 2006), there is a compelling biological basis for more severe effects once the compound passes through the placenta. Given greater fetal and neonatal vulnerability, these aforementioned results raise quite reasonable concerns for parents and warrant research utilizing proximity to pesticides as an exposure indicator when biological samples are unavailable (Harnly et al. 2005).

Finally, Burns et al. (2015) include several misrepresentations of the scientific literature. For example, a review of behavioral impacts of chlorpyrifos expsure in rodent studies (Williams and DeSesso 2014) is cited to support the argument that doses reaching pregnant women neighboring agricultural fields are too low to cause harm to the human fetus. It is unclear how Burns et al. (2015) extrapolated to draw this conclusion or what assumptions they made regarding comparability of rodent versus human dosing.

Burns et al. (2015) also reference work by Ward et al. (2006), who used a spatial model to predict household carpet dust levels of agricultural pesticides. Ward et al. (2006) reported, “Increasing acreage of corn and soybean fields within 750 m of homes was associated with significantly elevated odds of detecting agricultural herbicides [in house dust] compared with homes with no crops within 750 m.” Yet Burns et al. (2015) state to the contrary, “Proximity to agricultural pesticide application has not been found to translate to corresponding levels of the pesticide in household dust (Curwin et al. 2005; Fenske et al. 2002; Ward et al. 2006).” Other results from these cited articles also are misrepresented: Fenske et al. (2002) reported higher chlorpyrifos in house dust for homes in closer proximity (*p* = 0.01), and Curwin et al. (2005) detected higher levels in farm homes than in nonfarm homes.

## References

[r1] Alarcon WA Calvert GM Blondell JM Mehler LN Sievert J Propeck M 2005 Acute illnesses associated with pesticide exposure at schools. JAMA 294 4 455 465; 10.1001/jama.294.4.455 16046652

[r2] Burns CJ Cohen SZ Lunchick C 2015 Neurodevelopmental disorders and agricultural pesticide exposures. Environ Health Perspect 123 4 A79; 10.1289/ehp.1409124 25831272 PMC4384206

[r3] Chen J Kumar M Chan W Berkowitz G Wetmur JG 2003 Increased influence of genetic variation on PON1 activity in neonates. Environ Health Perspect 111 11 1403 1409; PMID:12928148 10.1289/ehp.6105 PMC1241633

[r4] Curwin BD Hein MJ Sanderson WT Nishioka MG Reynolds SJ Ward EM 2005 Pesticide contamination inside farm and nonfarm homes. J Occup Environ Hyg 2 7 357 367; 10.1080/15459620591001606 16020099

[r5] Fenske RA Lu C Barr D Needham L 2002 Children’s exposure to chlorpyrifos and parathion in an agricultural community in central Washington State. Environ Health Perspect 110 5 549 553; PMID: . 12003762 10.1289/ehp.02110549 PMC1240847

[r6] Furlong CE Holland N Richter RJ Bradman A Ho A Eskenazi B 2006 PON1 status of farmworker mothers and children as a predictor of organophosphate sensitivity. Pharmacogenet Genomics 16 3 183 190; PMID: . 16495777 10.1097/01.fpc.0000189796.21770.d3

[r7] Gunier RB Ward MH Airola M Bell EM Colt J Nishioka M 2011 Determinants of agricultural pesticide concentrations in carpet dust. Environ Health Perspect 119 7 970 976; 10.1289/ehp.1002532 21330232 PMC3222988

[r8] Harnly M McLaughlin R Bradman A Anderson M Gunier R. 2005 Correlating agricultural use of organophosphates with outdoor air concentrations: a particular concern for children. Environ Health Perspect 113 9 1184 1189; 10.1289/ehp.7493 16140625 PMC1280399

[r9] Huen K Bradman A Harley K Yousefi P Boyd Barr D Eskenazi B 2012 Organophosphate pesticide levels in blood and urine of women and newborns living in an agricultural community. Environ Res 117 8 16; 10.1016/j.envres.2012.05.005 22683313 PMC4309544

[r10] Lee SJ Mehler L Beckman J Diebolt-Brown B Prado J Lackovic M 2011 Acute pesticide illnesses associated with off-target pesticide drift from agricultural applications: 11 states, 1998–2006. Environ Health Perspect 119 8 1162 1169; 10.1289/ehp.1002843 21642048 PMC3237344

[r11] Shelton JF Geraghty EM Tancredi DJ Delwiche LD Schmidt RJ Ritz B 2014 Neurodevelopmental disorders and prenatal residential proximity to agricultural pesticides: the CHARGE study. Environ Health Perspect 122 10 1103 1109; 10.1289/ehp.1307044 24954055 PMC4181917

[r12] Ward MH Lubin J Giglierano J Colt JS Wolter C Bekiroglu N 2006 Proximity to crops and residential exposure to agricultural herbicides in Iowa. Environ Health Perspect 114 6 893 897; 10.1289/ehp.8770 16759991 PMC1480526

[r13] Williams AL DeSesso JM 2014 Gestational/perinatal chlorpyrifos exposure is not associated with autistic-like behaviors in rodents. Crit Rev Toxicol 44 6 523 534; 10.3109/10408444.2014.907772 24861450

[r14] Wofford P Segawa R Schreider J Federighi V Neal R Brattesani M. 2014 Community air monitoring for pesticides. Part 3: using health-based screening levels to evaluate results collected for a year. Environ Monit Assess 186 3 1355 1370; 10.1007/s10661-013-3394-x 24370859

